# Micro flow photochemical synthesis of Ca‐sensitive fluorescent sensor particles

**DOI:** 10.1002/elsc.202100023

**Published:** 2021-06-04

**Authors:** Klaus‐Peter Kronfeld, Thomas Ellinger, Johann Michael Köhler

**Affiliations:** ^1^ Department of Physical Chemistry and Microreaction Technology Technical University Ilmenau Ilmenau Germany; ^2^ Blink AG Jena Germany

**Keywords:** calcium determination, fluorescence sensing, hydrogel particles, microfluidics, Rhod‐5N

## Abstract

Fluorescence probes have widely been used for detecting and imaging Ca^2+^‐enriched parts of cells but more rarely for quantitative determination of concentrations. In this study we show how this can be achieved by a novel approach using hydrogel particles. In a microfluidic co‐flow arrangement spherical droplets were generated from an aqueous solution of acrylamide, *N,N*'‐methylenebisacrylamide crosslinker and photoinitiator and subsequently photo‐cured in situ yielding gel particles in a sub millimeter range. These particles were separated, dried under reduced pressure and re‐swollen in water containing Rhod‐5N tri potassium salt as calcium ion selective fluorescence probe. After that the particles were dried again and stored for further investigations. Upon exposure of dried particles to calcium chloride solutions they swell and take up Ca^2+^‐ions forming a strong fluorescing complex with Rhod‐5N. Thus, fluorescence intensity increases with calcium ion concentration. Up to ca. 0.50 mM the enhancement effect is strong and then becomes considerably weaker. The intensity‐concentration‐dependence is well described by an equation derived from the equilibrium of the formation of a 1:1 Ca^2+^:Rhod‐5N complex. The particles allow for a fast optical determination of Ca^2+^‐concentrations up to 0.50 mM in analyte volumes down to below 10 μL.

**Abbreviations**
*c*_A_
: equilibrium‐concentration of analyte (Ca^2+^)
*c*_A0_
: initial concentration of analyte (Ca^2+^)
*c*_R_
: equilibrium‐concentration of Rhod‐5N
*c*_R0_
: initial concentration of Rhod‐5N
*c*_RA_
: equilibrium‐concentration of Rhod‐5N ‐ Ca^2+^‐complex
ID: inner diameter (of a tube)
*I*_max_
: fluorescence intensity of the complexated dye (at high Ca^2+^; *c*_RA_ = *c*_R0_, *c*_R _= 0)
I_min_
: fluorescence intensity of the non‐complexated dye (without Ca^2+^; *c*_RA_ = 0, *c*_R _= *c*_R0_)
*K*_d_
: complex dissociation constant
*n*_RL_
: mol‐quantity of Rhod‐5N in a particle
PAA: polyacrylamide
*V*_0_
: volume of the re‐dried, dye‐loaded sensor particle
*V*_L_
: volume of the dye‐loaded, swollen particle
*V*_M_
: particle volume at measurement time
*V*_R_
: volume of the raw, dry particle

## 1 INTRODUCTION

The local determination of calcium concentration is of large interest for cell technology and tissue physiology [1]. It is well known, that Ca‐sensitive dyes can be applied for the local measurement of calcium concentrations and for the characterization of gradients in the calcium content [2], but the dissolved sensor dyes can interfere with the physiological processes in the investigated biological objects. Therefore, micro particles are of interest with the Ca‐sensitive dyes fixed in a polymer matrix.

The application of micro and nanoparticles as primary transducers for local chemical sensing is a universally usable concept. It was already applied for determination of different metal ions, among them Al^3+^ and Zn^2+^ by using the fluorescence of *N,N*’‐ethylenebis(salicylimine) [3]. Luminescence‐active inorganic nanoparticles have been applied for the detection of ions like Fe^3+^ and Hg^2+^ [4], for example. A quantum dot‐based (QD) generation of a Ca^2+^‐related signal succeeded by coupling QDs with a Rhodamine‐Fluorescence‐Resonance‐Energy‐Transfer System [5]. Despite inorganic nanoparticles, organic particles can be used for the sensing of metal ions, too. Thus, it was shown that Sn^2+^ can be detected by salicylaldehyde‐modified organic nanoparticles [6]. Polymer microparticles have been developed for the determination of pH and for the in situ measurement for oxygen concentration [7]. Such sensors are well suited for the chemical characterization of micro droplets and have been used in toxicological studies in the micro segmented flow technology [8].

Molecular indicator dyes are widely used in chemical analytics for a long time. But their application in homogeneous phase for biological systems is limited by the risk of incompatibilities between physiological activity of cells or tissues and the dye molecules dispersed in solution [9, 10]. On the one hand side, dyes can be toxic and inhibit the growth of cells and microbial cultures. On the other hand they can be degraded by metabolization processes. Both effects can be reduced if the indicator molecules are immobilized inside a permeable polymer matrix. This strategy is frequently applied in test strips and films and is usable for sensor particles, too. Newer techniques of immobilization comprise trapping of sensor dyes in nanopores, which are opened by target molecules via specific reaction chains [11, 12] or intercalating them between DNA strains [13]. In this way toxins and proteins have been measured sensitively using the fluorescence [11] and redox [12, 13] characteristics of rhodamine B and methylene blue, respectively.

Crosslinked polyacrylamide had been found to be well suited as matrix material for sensor purposes [14]. Polyacrylamide hydrogel particles can be generated in high quality by a continuous flow process with in situ photo‐polymerization [15]. Droplets of polymer mixture in aqueous solution were generated by co‐flow arrangements or flow‐focusing techniques by injection of the reaction mixture into a flow of an inert carrier liquid. The solidification of droplets is then started by a short‐time UV‐exposure initiating a radical polymerization. This technique is applicable for different polymer and hydrogel materials [16] and is also applicable for the generation of hydrogel components for hierarchically constructed particles for sensing and catalytic applications [17].

**PRACTICAL APPLICATION**

Rhod‐5N, a calcium sensitive fluorescence dye, is because of its moderate complexation strength suited for measurement of physiological calcium concentrations. The dye can be immobilized in hydrogel spheres consisting of crosslinked polyacrylamide, which are stable in dried state and ready to use. Immersed in analyte solution those particles swell and show calcium dependent fluorescence enhancement. Particles can be measured in situ or after separation, which can be useful if the analyte is colored or opaque. Since the fluorescence intensity over a wide Ca^2+^ concentration range can be described by an equation with only three parameters, calibration is easy. Using microparticles also allows for automation and multiplexing when they are loaded with different probes.

In a recent paper [18], we have shown the application of PAA particles loaded with lucigenin for determination of chloride by dynamic fluorescence quenching. Here, this strategy is applied for the synthesis of calcium‐sensitive hydrogel particles incorporating the dye Rhod‐5N and thus extended for the determination of Ca^2+^ by the formation of a fluorescent ground state complex.

## 2 MATERIALS AND METHODS

Methods and materials are widely described in ref. [18].

### 2.1 Materials

Silicone oil 500 cSt was used as continuous flowing phase inside the micro flow reactor. Acrylamide:*N,N*'‐methylenebisacrylamide 19:1 was used as monomer for particle synthesis dissolved in deionized water. The photoinitiator lithium 2,4,6‐trimethylbenzoyl‐phenylphosphinate (Li‐TPO) was synthesized according to literature [19]. Rhod‐5N tri potassium salt was purchased from Invitrogen; analytical grade n‐heptane calcium chloride and sodium dodecylsulfate were used as supplied.

### 2.2 Methods

#### 2.2.1 Synthesis of polymer particles

**FIGURE 1:**
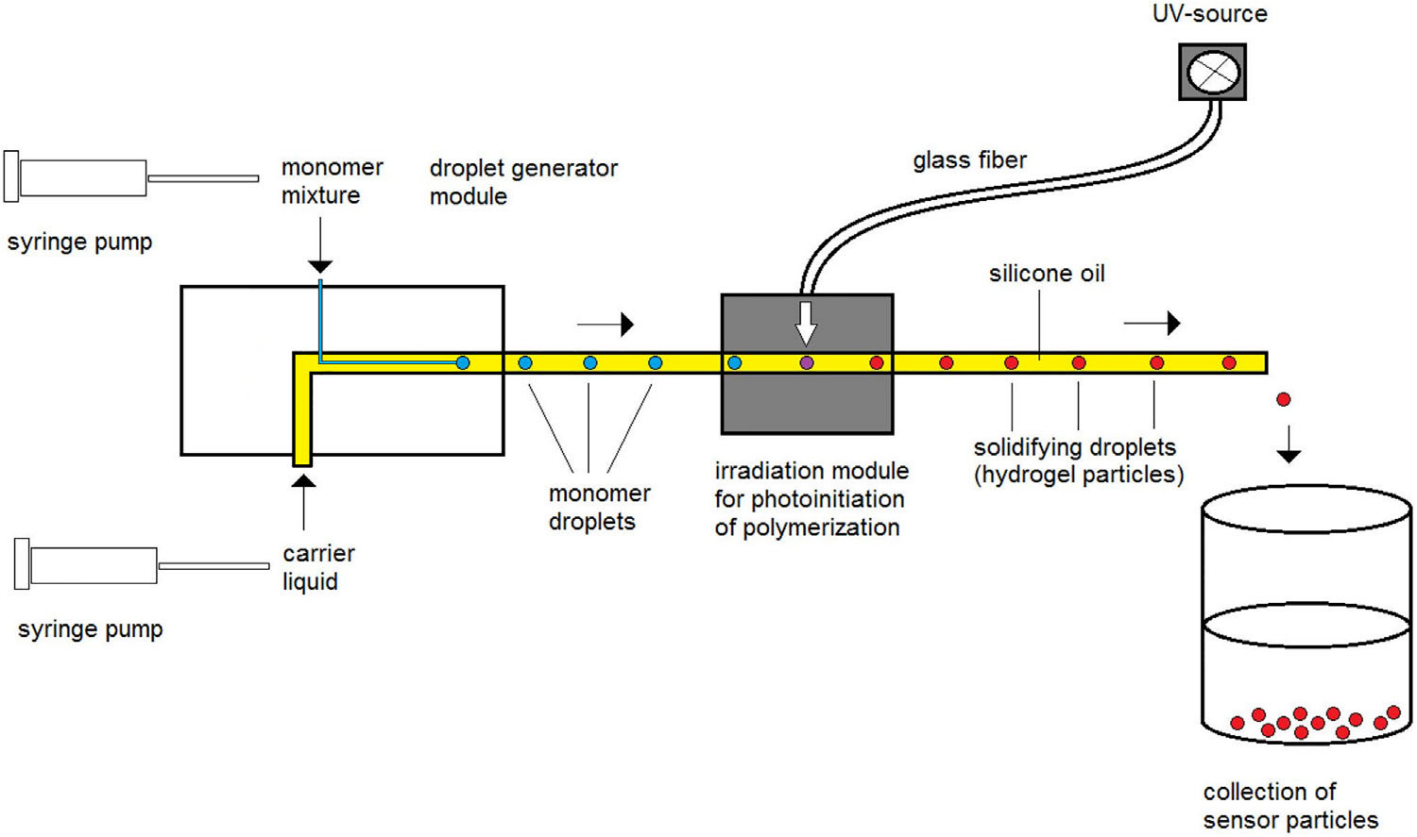
Experimental set‐up for fluidic generation of hydrogel sensor particles

PAA hydrogel particles have been prepared from the monomer/crosslinker/initiator solution (450 mg Acrylamide/*N,N*'‐methylenebisacrylamide, 7.5 mg Li‐TPO in 1.5 mL deionized water) using the co‐flow‐arrangement described in [18] consisting of two syringe pumps, a droplet generation module and an irradiation module, see Figure 1. Inside the droplet generation module a continuous silicon oil phase flows in a 1.5 mm (ID) wide glass tube. In the center of this tube the monomer solution is introduced through a narrow inner capillary forming spherical droplets, which are conveyed further with the oil stream. In order to produce particles of different sizes two sets of parameters were applied.

Set 1: capillary ID 600 μm (metal), flow rate 200/30 μL/min (continuous/monomer)
Set 2: capillary ID 250 μm (fused silica), flow rate 400/20 μL/min (continuous/monomer)

Instantly after droplet generation the still liquid particles run through the irradiation unit being exposed to near UV‐light. Thereby the photoinitiator decomposes to free radicals, which initiate polymerization leading to solidification of the particles. Irradiation is performed at 17% and 33% of the maximum intensity of the lamp for set 1 and 2, respectively.

#### 2.2.2 Finishing of the sensor particles

The particle suspension was poured into an empty SPE‐cartridge with a PE‐frit. By applying gentle vacuum the main part of the oil was removed from the PAA particles. Finally, ultimate oil traces were removed by washing three times with n‐heptane. Thus cleaned particles were spread on a petri dish and left there for about 30 min on the air for evaporation of the excess heptane. Subsequently, they have been dried in vacuo and loaded with 100 mg/L (1.11•10^−4^ M) Rhod‐5N in water as described earlier [18]. After re‐drying they are still swellable having a shelf life of at least 3 months.

#### 2.2.3 Spectrometry and microscopy

Absorption spectra of solutions have been recorded by a spectrometer Specord 200 (Analytic Jena), uncorrected emission spectra by a fluorimeter JASCO FP‐8300. Optical measurement of particles has been carried out using a fluorescence microscope Zeiss Axioplan 2. Excitation was managed to be in the green spectral region (510‐560 nm) by means of Zeiss Filter Set 14 (item number 488014‐0000‐000). Light intensity was held constant during one series of measurements. A single particle was followed up by placing the dry particle in the indentation of a glass slide under the microscope and taking an initial photo. After adding of analyte liquid the focus was re‐adjusted and further photos were taken in short time periods to monitor both fluorescence intensity and particle size dependent on time and quantity of CaCl_2_ added. Images have been processed by using graphic software ImageJ.

## 3 RESULTS AND DISCUSSION

### 3.1 Particle sizes and swelling process

**FIGURE 2:**
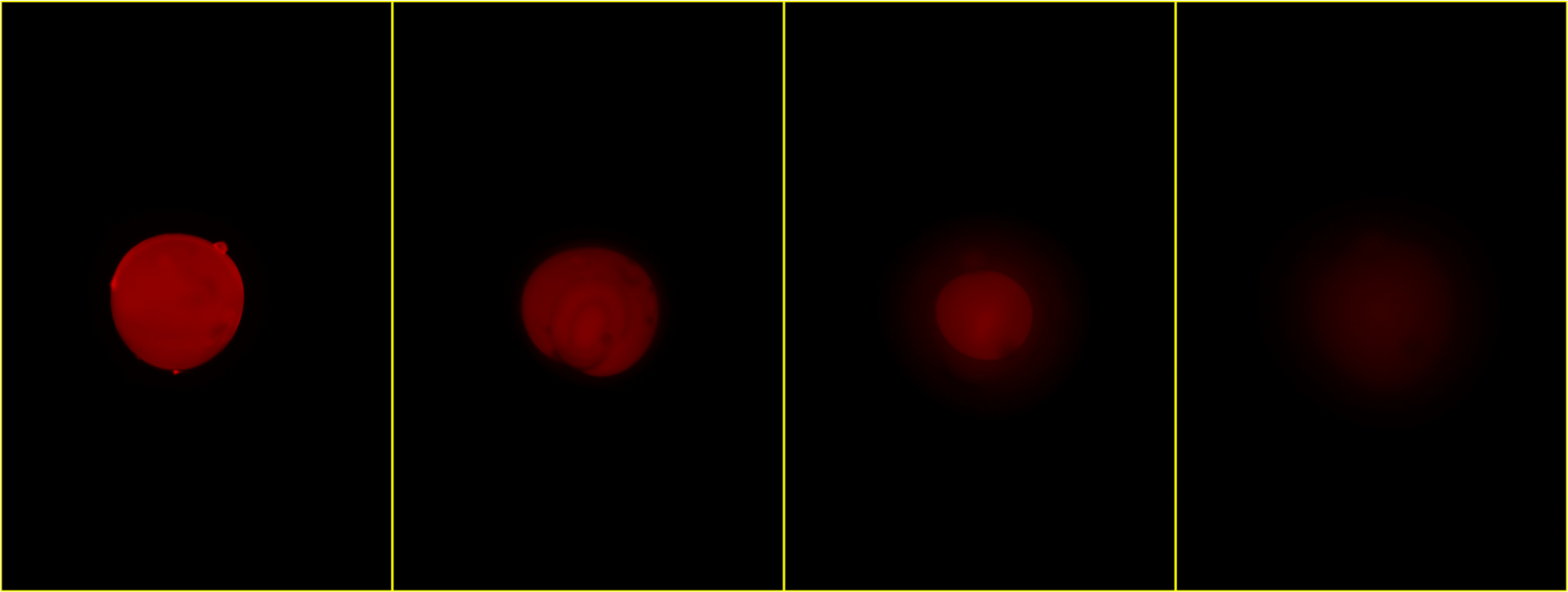
Fluorescence images of one Rhod‐5N‐containing PAA sensor particle (set 1) in dry state and after immersion in water for 22, 280, and 630 s

**FIGURE 3:**
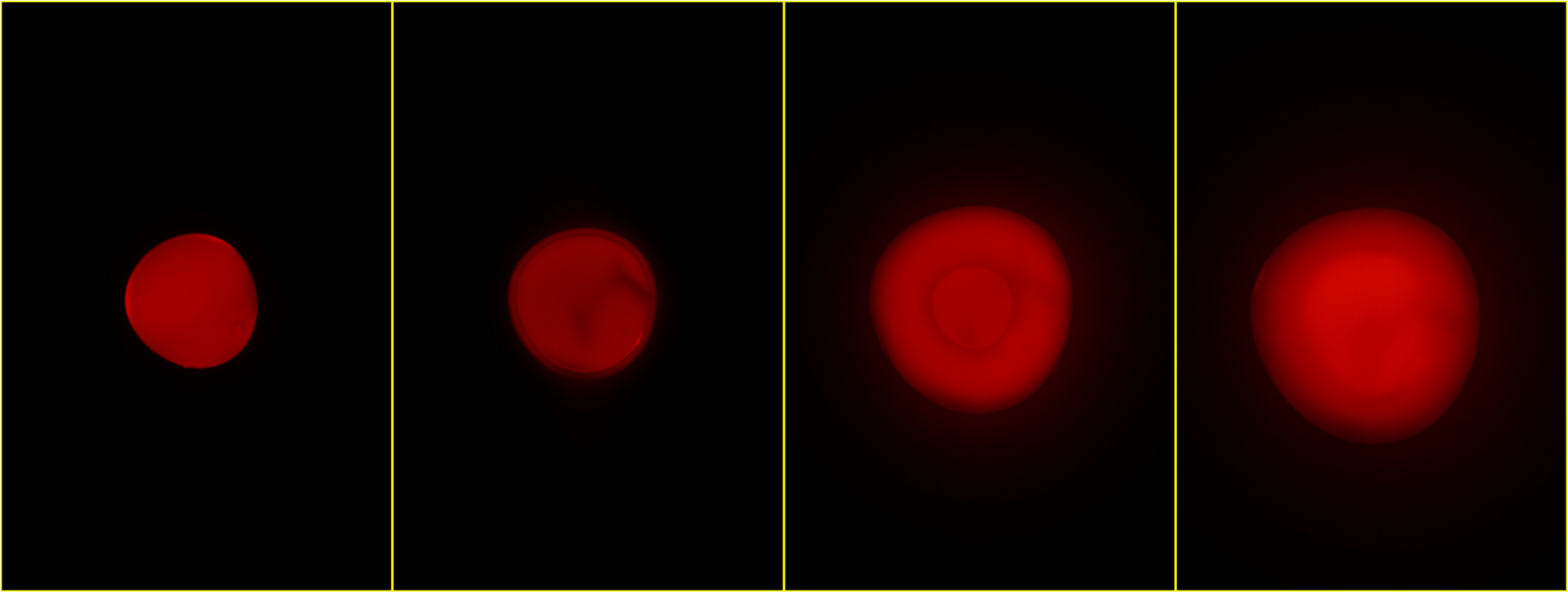
Fluorescence images of one Rhod‐5N‐containing PAA sensor particle (set 1) in dry state and after immersion in 5 mM CaCl_2_ solution for 14, 298, and 618 s

**FIGURE 4:**
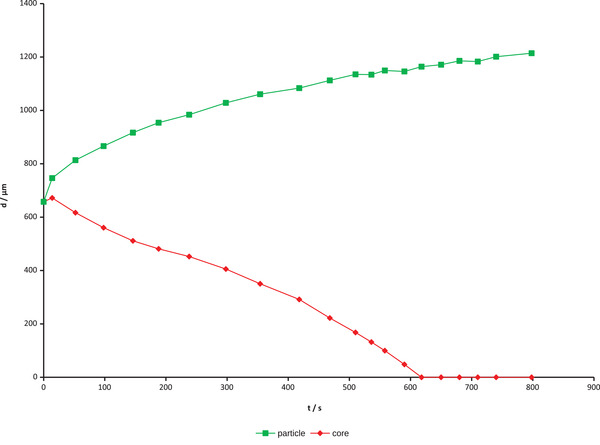
Swelling behavior of one dried Rhod‐5N‐loaded hydrogel particle (set 1) in 5 mM CaCl_2_ solution (■ particle diameter, ♦ un‐swollen particle core)

**FIGURE 5:**
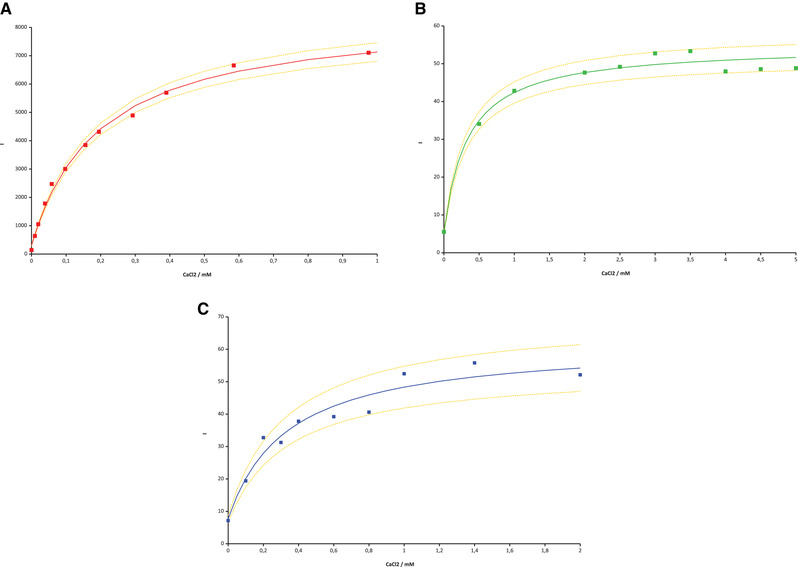
Plots of fluorescence intensity vs. Ca^2+^ concentration in solution (A) and hydrogel particles of set 1 (B) and set 2 (C), regression curves according to Equation (1) – dashed lines: error limits for 1.5‐fold standard deviation; (A) *λ*_ex_ 553 nm, *λ*_meas_ 571 nm, [Rhod‐5N] = 2.78•10^−6^ M; (B, C) measured intensity (gray values) instantly after main swelling process, [Rhod‐5N] ≈ 1.2•10^−4^ M (B), 1.5•10^−4^ M (C)

Sizes of Rhod‐5N loaded dry particles prepared according to set 1 and 2 are 680 and 350 μm, respectively, with a narrow size distribution (standard deviation 1.6% and 3.2%) as shown in Table 1. Upon immersion in water (Figure 2) and 5 mM calcium chloride solution (Figure 3) particles begin to swell and also change their fluorescence behavior over time. In dry state at the beginning of the swelling process they show a rather strong fluorescence. This can be attributed to a lack of non‐radiative deactivation paths of the excited dye in a rigid environment. As water moves in the polymer network widens and Rhod‐5N molecules become flexible. Thus, in pure water the fluorescence almost disappears along the diffusion front (Figure 2). Contrary, in the presence of excess Ca^2+^ a complex is formed, which fluoresces similar like the yet non swollen particle core (Figure 3) making the front more difficult to identify. It is obvious that quantitative fluorescence measurements have to be taken after the core has resolved completely. Typically, this is accomplished within 2‐3 min for the smaller particles and 10 min for the larger ones. After that particle size changes less than 7% and fluorescence intensity less than 25% within the next 2‐3 min. In this study those measurements were taken a few seconds after the core disappeared. Figure 4 shows a plot of particle size vs. time of one of the larger particles in 5 mM CaCl_2_ solution. While the overall particle size grows with a decreasing rate, the non‐swollen core vanishes more or less linearly with time, like we already observed earlier [18]. Fluorescence images (Figures 2 and 3) also show no significant fluorescence outside the particles, that is, no loss of dye into the environment.

**TABLE 1:** Particle sizes of samples from two preparation‐batches

**Batch**	**Diameter in μm of Rhod‐5N loaded particles above: dry, below: swollen (at time of measurement)**	**Mean value [μm]**	**Standard deviation**
Set 1 capillary 600 μm flow 30/200 μL/min lamp‐intensity 17%	677, 670, 680, 661, 683, 683, 678, 682, 701 1184, 1135, 1235, 1171, 1153, 1142, 1170, 1104, 1182	679 1164	1.6% 3.2%
Set 2 0.15% SDS added capillary 250 μm flow 20/400 μL/min lamp‐intensity 33%	372, 340, 348, 336, 353, 330, 364, 349, 343 548, 529, 535, 624, 611, 589, 643, 612, 647	348 593	3.8% 7.7%

### 3.2 Model for Ca^2+^‐determination by formation of a fluorescent complex with Rhod‐5N

Rhod‐5N tri potassium salt is a commercially available fluorescence probe, which is well suited for the determination of physiologically relevant Ca^2+^‐concentrations [20, 21] because of its moderate complexation constant with Ca^2+^ and its insensibility towards Mg^2+^ [20, 22]. Rhod‐5N is composed of a fluorophore entity (rhodamin like) and a complexing BAPTA‐derived part (BAPTA: 1,2‐bis(o‐aminophenoxy)ethane‐*N,N,N′,N*′‐tetraacetic acid), which share one common benzene ring (cf. structure).


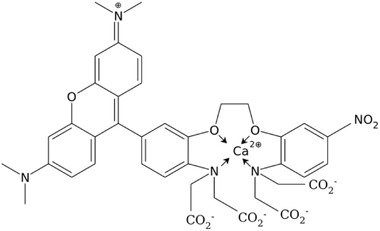


Structure of Rhod‐5N with complexed calcium ion

Without complexing ions it shows only weak fluorescence due to fluorescence quenching of the rhodamine part by electron transfer from the free electron pair at the adjacent nitrogen atom [21]. In the presence of Ca^2+^‐ions a complex is formed at the BAPTA‐entity with electron pairs at nitrogen and oxygen atoms fixed to calcium. Thereby electron transfer is hindered. Additionally, the complex has less motional degrees of freedom than the un‐complexed molecule, which would form another deactivation path of the exited state. Thus, fluorescence remains the favored way to dissipate excess energy.

The equilibrium constant for the formation of a 1:1 complex can be expressed by

$$\;K_{d}=\frac{c_{R}c_{A}}{c_{RA}}\;=\frac{\left(c_{R0}-c_{RA}\right)\left(c_{A0}-c_{RA}\right)}{c_{RA}}$$

leading to the equation for calculation of *c_RA_*

$$c_{RA}^{2}-\left(c_{R0}+c_{A0}+K_{d}\right)c_{RA}+c_{R0}c_{RA}=0$$

with the solution:

$$\;c_{RA}=\frac{c_{R0}+c_{A0}+K_{d}}{2}*\left\{1-\sqrt{1-\frac{4c_{R0}c_{A0}}{{\left(c_{R0}+c_{A0}+K_{d}\right)}^{2}}}\right\}$$

Since the second term beneath the square root is small compared to 1, the root can be developed into a Taylor series neglecting all members greater than 1, resulting in:

$$c_{RA}\approx \frac{c_{R0}c_{A0}}{c_{R0}+c_{A0}+K_{d}}$$

and hence for *c_R_* = *c_R0_* – *c_RA_*

$$c_{R}\approx \frac{c_{R0}\left(c_{R0}+K_{d}\right)}{c_{R0}+c_{A0}+K_{d}}$$

Provided the fluorescence intensities are proportional to the concentrations the sum‐intensity follows from the (weak) one of Rhod‐5N (*c*_R_) and the (strong) one of the complex (*c*_RA_):

$$I=I_{max}\frac{c_{RA}}{c_{R0}}+I_{min}\frac{c_{R}}{c_{R0}}$$

With the equations for *c*_R_ and *c*_RA_ it eventually follows:

(1) $$I\approx I_{min}+\left(I_{max}-I_{min}\right)\frac{c_{A0}}{c_{A0}+K_{d}+c_{R0}}.$$

If *I*_min_ is precisely known this equation can be linearized by subtracting *I*_min_ from both sides and forming the reciprocal:

(2) $$\begin{array}{c} {\left(I-I_{min}\right)}^{-1}\approx {\left(I_{max}-I_{min}\right)}^{-1}*\left\{1+\left(K_{d}+c_{R0}\right)*c_{A0}^{-1}\right\}. \end{array}$$

However, by plotting 1/(*I*‐*I*_min_) versus 1/*c*_A0_ lower I‐ and c‐values are overestimated in the linear regression, and *I*_min_ is not considered an ordinary measurement. Whereas nonlinear regression of Equation (1) by the least‐square‐method provides more adequate results for all parameters (*I*_min_, *I*_max_, *K*_d_ + *c*_R0_) and is therefore applied subsequently.

Figure 5 shows fluorescence enhancement measured in solution and hydrogel particles, respectively, with curves fitted to Equation (1). In solution *K*_d_ + *c*_R0_ equals 1.96•10^−4^ M. Taking c_R0_ into account, for *K*_d_ 1.94•10^−4^ M is obtained (manufacturer's data [17] 3.2•10^−4^ M). For the hydrogel particles by the same way apparent values for *K*_d_ + c_R0_ of 3.38•10^−4^ M for the smaller particles and 3.34•10^−4^ M for the larger particles are obtained. However, in this case it must be considered that the analyte concentration at measurement time *c*_AM_ is lower than the nominal concentration *c*_A0_ in the surrounding solution due to the dilution effect of the gel material. It follows:

(3) $$c_{AM}=\left(V_{M}-V_{0}\right)/V_{M}*c_{A0}=\left(1-V_{0}/V_{M}\right)*c_{A0}\;.$$

Also, the concentration of Rhod‐5N at measurement time is different from the concentration used for loading the particles. Upon loading a particle takes up *n*_RL_ = (*V*_L_–*V*_R_) *c_R0_ mol of dye remaining unchanged until measurement. Thus, dye concentration at measurement is

(4) $$c_{RM}=n_{RL}/V_{M}=\left(V_{L}-V_{R}\right)/V_{M}*c_{R0}.$$

Replacing *c*_A0_ and *c*_R0_ in Equation (1) by *c*_AM_ and *c*_RM_, respectively, leads to:

$$I\approx I_{min}+\left(I_{max}-I_{min}\right)\frac{c_{A0}}{c_{A0}+fK_{d}+gc_{R0}}$$

with

$$f=V_{M}/\left(V_{M}-V_{0}\right);g=\left(V_{L}-V_{R}\right)/\left(V_{M}-V_{0}\right)$$

Thus, regression of *I* versus *c*_A0_ values yields the parameter *r* = (f*K_d_ + g*c_R0_) rather than (*K*_d_ + *c*_R0_). Hence, *K*_d_ can be calculated:

(5) $$K_{d}=\left(V_{M}-V_{0}\right)/V_{M}*r-\left(V_{L}-V_{R}\right)/V_{M}*c_{R0}.$$

The first corrective factor (V_M_‐V_0_)/V_M_ in Equation (5) is obtained directly from the dry and swollen particle sizes from Table 1 and is 0.80 for both particle batches. The second factor (*V*_L_‐*V*_R_)/*V*_M_ was determined separately for the smaller particles to be 1.33 and estimated 1.08 for the larger ones. Thus, *K*_d_ can be calculated:

Larger particles (set 1):

$$K_{d}=\left(0.80*3.34-1.08*1.11\right)•10^{-4}M=1.47•10^{-4}M$$

Smaller particles (set 2):

$$K_{d}=\left(0.80*3.38-1.33*1.11\right)•10^{-4}M=1.23•10^{-4}M$$

Both values are in rather good agreement with each other, with the value of 1.94•10^−4^ M determined for solution and also with a reported value of 1.40•10^−4^ M [22].

### 3.3 Application of the model for Ca^2+^ measurement and estimation of errors

#### 3.3.1 Theoretical estimation of errors

It starts with the question how the fluorescence intensity “I” at a given analyte concentration *c*_A0_ is affected when using different sensor particles of the same batch due to fluctuations in particle size. It can be calculated (see supporting information for details) that for a *c*_A0_ of 0.5 mM the observed intensity is expected to diverge from the most probable value by –1.3% to +1.1% for the larger particles and –5.7% to +4.3% for the smaller.

In reality the problem is inversed: The measured intensity is given and the concentration sought. When intensity is uncertain by about ±1.2% (set 1) or ±5.0% (set 2) one calculates in return for c_A0_ using Equation (1) and values of *I*_min_, *I*_max_, (*K*_d_ + *c*_R0_) directly from regression uncertainties for *c*_A0_ of approximately ±5.2% (set 1) and ±19.1% (set 2).

#### 3.3.2 Practical measurement data

As can be seen from the reading points and the curves of Figure 5B,C; Equation (1) represents a good approximation for the intensity‐concentration‐relationship. It also shows that calcium determination up to ca. 0.50 mM is technically feasible. At higher concentrations the further growth of the fluorescence signal is too small for accurate measurements. In such cases the analyte may be diluted prior to measurement. For practical use for a given batch of particles the parameters of Equation (1) can be calculated by a 3‐point calibration, for example, at 0.0, 0.3, and 1.0 mM. Table 2 shows absolute and relative errors for the measurements from Figure 5C, with three pairs of values (bold rows) taken for calibration. The relative errors of readings between the calibration points are sometimes high (up to about 60%). This is basically in line with the theoretical estimation. It is even worse since the parameters obtained by calibration are less reliable than those from a full regression. The problem should be overcome by using a greater number of particles of the same batch in one measurement. For this purpose smaller particles are beneficial and also for speeding up cycle times. From Table 2 a detection limit can also be estimated: Provided intensity at zero Ca^2+^ (7.2) is uncertain by 30%, only intensities above 9.4 can be attributed to a non‐zero calcium concentration. This value corresponds to a Ca^2+^ of about 0.02 mM derived from calibration, which is the limit.

**TABLE 2:** Errors observed at Ca^2+^‐determination after 3‐point calibration using Rhod‐5N loaded hydrogel particles of batch set 2

Intensity	[Ca^2+^]	[Ca^2+^]_calc_	Error	
[a. u.]	[mM]	[mM]	abs.	rel.
**7.2**	**0**			
19.4	0.1	0.122	+0.022	+22.0%
32.7	0.2	0.327	+0.127	+63.5%
**31.3**	**0.3**			
37.8	0.4	0.440	+0.040	+10.0%
39.2	0.6	0.476	‐0.124	‐20.7%
40.6	0.8	0.514	−0.286	−35.8%
**52.5**	**1.0**			

## 4 CONCLUDING REMARKS

The investigations show that the determination of calcium ions via a fluorescing complex succeeds by the application of hydrogel sensor particles. Such particles can be generated with narrow size distribution by a continuous micro flow process using an in situ photo polymerization step. The particles can be dried and reactivated with the fluorescence probe in an aqueous environment. After re‐drying they still can take up more than 450% of their initial volume of analyte solution during swelling. The diameter of about 0.3 to 0.7 mm (dry state) allows for single particle measurements on sampling volumes down to the 10 μL level within 2 to 10 min response time. The fluorescence intensity shifts considerably at the physiological Ca^2+^ concentration range between 0.0 and 1.0 mM following a plain mathematical function.

Yet for practical application the errors are sometimes too high, which is mainly brought about by the uncertainty of the particle volume at measurement time. Besides the use of a manifold of particles an even sharper size distribution and an optimized timing of measurement should bring improvements.

Their quality and the sensing properties show that polyacrylamide hydrogel particles are powerful for miniaturized transduction of chemical into optical signals. Combined with adequate analytical dyes this method is particularly suited for measurement of electrolytes (cat‐ and anions) and small molecules (urea, glucose) in small sample volumes. For example, it could be applied for optical read‐out in micro cuvettes, micro capillary slits or in microliter fluid segments. Using micro particles with sensitivity for distinct components simultaneously complex composed mixtures can be characterized. Important future applications are seen in fast medical point‐of care diagnostics and analyses in the environment.

## CONFLICT OF INTEREST

The authors have declared no conflict of interest. The manuscript neither contains experiments using animals nor are human studies involved in this work.

## Supporting information

Supporting information

## Data Availability

The data that support the findings of this study are available from the corresponding author upon reasonable request.
