# Supplementary material for: Micro flow photochemical synthesis of Ca‐sensitive fluorescent sensor particles
Source: Eng Life Sci. 2021 Jun 4;21(8-9):518–26. doi: 10.1002/elsc.202100023 (PMC8456324; doi:10.1002/elsc.202100023)
Supplement: Supplementary file 1 — Supporting information [file ELSC-21-518-s001.pdf]

Additional Abbreviations:

d_0, diameter of the re-dried, dye-loaded sensor particle
d_M, particle diameter at measurement time
φ_F, fluorescence quantum yield of the Rhod-5N-calcium-complex
I_{abs}, absorbed light intensity
I_0, intrinsic light intensity of the lamp
E, absorbance (at the center) of the particle
ε, (decadic) molar absorptivity of the complex ($\varepsilon_{570} \approx 76000\ M^{-1}cm^{-1}$)

Theoretical estimation of errors

Provided eq. (1) is an adequate model the effect of particle sizes on fluorescence intensity can be estimated. Thereby, sizes at every state of preparation, i.e. after synthesis, loading, re-drying and measurement have to be considered. These figures influence the actual concentrations c_{RM} and c_{AM} in the particles and hence light absorption and emission (I_{min}, I_{max}). For a basic approach the influence of I_{min} can be neglected, because it mostly contributes only a small value.

a) c_{RM} is given by eq. (4). It can be assumed that (V_L-V_R) and V_M are not independent from each other since the more the particle swells at loading the more it should be swollen at measurement. Thus as a first approximation it can be assumed that the relative error in c_{RM} equals that in V_M that is three times that of d_M (because $V \sim d^3$). With $c_{R0} = 1.11•10^{-4}$ M one gets $c_{RM} = (1.20 \pm 0.12)•10^{-4}$ M for set 1 and $(1.48 \pm 0.34)•10^{-4}$ M for set 2, respectively.

b) c_{AM} is given by eq. (3). With $x = V_0/V_M$ it follows

$dc_{AM} = -dx * c_{A0}$ $\qquad$ $dc_{AM}/c_{AM} = -dx/(1-x) = - x/(1-x) * dx/x$

Since 1-x = 0.80 (see 3.2) it follows $dc_{AM}/c_{AM} = -0.25 * dx/x = -0.25 * d(V_0/V_M)/(V_0/V_M)$.

Thus the relative error of c_{AM} is 0.75 times the sum of those of d_0 and d_M. As an example, for a nominal concentration $c_{A0} = 5.00•10^{-4}$ M one gets $c_{AM} = (4.00 \pm 0.14)•10^{-4}$ M for set 1 and $(4.00 \pm 0.34)•10^{-4}$ M for set 2, respectively.

c) I_{max} is determined by the absorbed light as $I_{max} = \varphi_F*I_{abs}$. The exact calculation of I_{abs} is not trivial because of the spherical shape of the particles. For simplicity, a cubic shape is used as approximation, where absorption is constant over the particle's cross section: $I_{abs} = I_0*(1-10^{-E})$. Absorbance E is given by Lambert-Beer's law: $E = \varepsilon*c_{RM}*d_M = \varepsilon*c_{R0}*(V_L-V_R)/V_M*d_M$. Taking into account the considerations under a) it follows $E \sim d_M/V_M \sim 1/d^2_M$. Thus the relative error in absorbance is the double error in d_M. With mean particle sizes of 1164 µm and 593 µm one gets

$E = 1.061 \pm 0.068$ $\qquad$ $I_{max} = (0.913 +0.013 / -0.015)*I_0$ $\qquad$ for set 1, and

$E = 0.665 \pm 0.102$ $\qquad$ $I_{max} = (0.784 +0.045 / -0.058)*I_0$ $\qquad$ for set 2, respectively.

With these figures (mean value, lower and upper limit) one can calculate with eq. (1) values for the expected Intensity "I" (using the particular K_d and neglecting I_{min}). One finds

$I = (0.548 +0.006 / -0.007)*I_0\ \varphi_F$ $\qquad$ for set 1, and

$I = (0.468 +0.020 / -0.027)*I_0\ \varphi_F$ $\qquad$ for set 2, respectively.
